# Microcirculation and blood transfusions during sepsis: leukoreduced (LR) versus non-LR red blood cells

**DOI:** 10.1186/cc12307

**Published:** 2013-03-19

**Authors:** A Donati, E Damiani, R Domizi, C Scorcella, A Carsetti, S Tondi, R Castagnani, N Mininno, P Pelaia, C Ince

**Affiliations:** 1 Università Politecnica delle Marche, Ancona, Italy; 2 Academic Medical Center, Amsterdam, the Netherlands

## Introduction

Microcirculatory alterations during sepsis impair tissue oxygenation, which may be further worsened by anemia. Blood transfusions proved not to restore O_2 _delivery during sepsis [1]. The impact of storage lesions and/or leukocyte-derived mediators in red blood cell (RBC) units has not yet been clarified [2]. We compared the effects of leukoreduced (LR) versus nonLR packed RBCs on microcirculation and tissue oxygenation during sepsis.

## Methods

A prospective randomized study. Twenty patients with either sepsis, severe sepsis or septic shock requiring RBC transfusion randomly received nonLR (Group 1, *n = *10) or LR (Group 2, *n = *10) fresh RBCs (<10 days old). Before and 1 hour after transfusion, microvascular density and flow were assessed with sidestream dark-field imaging sublingually. Thenar tissue O_2 _saturation (StO_2_) was measured using near-infrared spectroscopy and a vascular occlusion test was performed.

## Results

**Figure 1:**
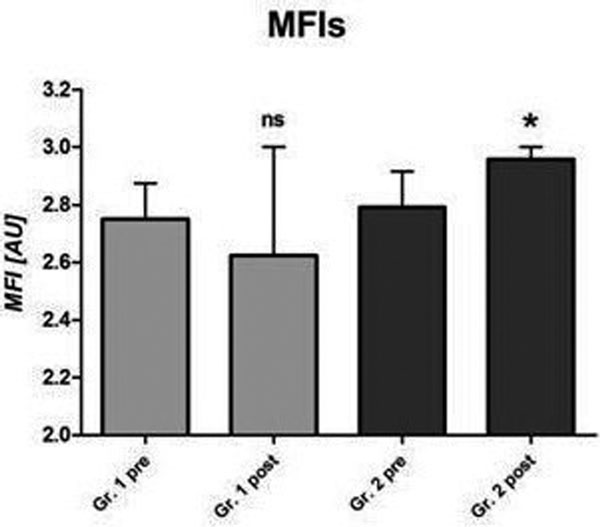
**Blood transfusion and microvascular flow**.

**Figure 2:**
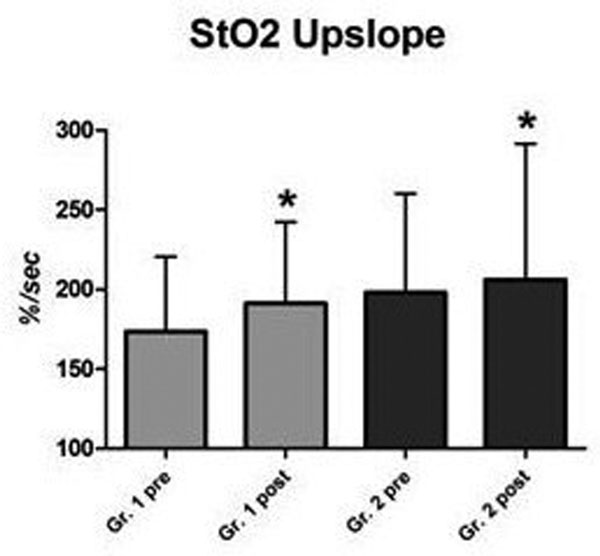
**Blood transfusion and microvascular reactivity**.

The De Backer score (*P *= 0.02), total vessel density (*P *= 0.08), perfused vessel density (*P *= 0.04), proportion of perfused vessels (*P *= 0.01), and microvascular flow index (*P *= 0.04, Figure 1) increased only in Group 2. The StO_2 _upslope (Figure 2) during reperfusion increased in both groups (*P <*0.05). In Group 1 the baseline StO_2 _and StO_2 _downslope during ischemia increased, probably reflecting a lower O_2 _consumption.

## Conclusion

Unlike nonLR RBCs, the transfusion of fresh LR RBCs seems to improve microvascular perfusion and might help to restore tissue oxygenation during sepsis.
